# Estimating the coverage of mental health programmes: a systematic review

**DOI:** 10.1093/ije/dyt191

**Published:** 2014-04-22

**Authors:** Mary J De Silva, Lucy Lee, Daniela C Fuhr, Sujit Rathod, Dan Chisholm, Joanna Schellenberg, Vikram Patel

**Affiliations:** ^1^Centre for Global Mental Health, London School of Hygiene and Tropical Medicine, London, UK, ^2^Department of Mental Health and Substance Abuse, World Health Organization, Geneva, Switzerland, ^3^Faculty of Infectious and Tropical Diseases, London School of Hygiene and Tropical Medicine, London, UK and ^4^Sangath, Alto-Porvorim, Goa, India

**Keywords:** mental health, programme coverage, evaluation, systematic review

## Abstract

**Background** The large treatment gap for people suffering from mental disorders has led to initiatives to scale up mental health services. In order to track progress, estimates of programme coverage, and changes in coverage over time, are needed.

**Methods** Systematic review of mental health programme evaluations that assess coverage, measured either as the proportion of the target population in contact with services (contact coverage) or as the proportion of the target population who receive appropriate and effective care (effective coverage). We performed a search of electronic databases and grey literature up to March 2013 and contacted experts in the field. Methods to estimate the numerator (service utilization) and the denominator (target population) were reviewed to explore methods which could be used in programme evaluations.

**Results** We identified 15 735 unique records of which only seven met the inclusion criteria. All studies reported contact coverage. No study explicitly measured effective coverage, but it was possible to estimate this for one study. In six studies the numerator of coverage, service utilization, was estimated using routine clinical information, whereas one study used a national community survey. The methods for estimating the denominator, the population in need of services, were more varied and included national prevalence surveys case registers, and estimates from the literature.

**Conclusions** Very few coverage estimates are available. Coverage could be estimated at low cost by combining routine programme data with population prevalence estimates from national surveys.

## Introduction

The large disparity between the number of people estimated to suffer from mental disorders and the proportion of those who receive adequate and appropriate treatment for these disorders, known as the ‘treatment gap’, is a core concern of the emerging discipline of global mental health.^1^ The median global treatment gap is estimated to be 32.2% for schizophrenia and 56.3% for depression.^2^ This has resulted in a dominant focus of current global mental health initiatives to scale up mental health services.^3–5^ In order to track the progress of regional, national and global efforts to reduce the treatment gap, evaluations of service need, utilization rates and programme coverage levels in populations are essential. These are all measures of the inverse of the treatment gap: treatment coverage.

Coverage can be conceived of as a one-dimensional concept such as a target for successful service scale-up of a particular intervention (such as vaccination rates in the under-five population), or as a multi-dimensional concept for moving towards universal access to health care. Multifaceted definitions of coverage include insurance against the risk of catastrophic health spending combined with access to an essential set of services.^6^

Intervention-specific or service coverage reflects the extent to which those in need of a health intervention get it. Individuals in need of a specific health service pass through a number of stages before they can receive the full therapeutic benefit of an intervention.^7^ Specifically, the intervention needs to be: (i) physically available; (ii) financially and geographically accessible; (iii) acceptable; (iv) used; and (v) delivered appropriately and effectively. Even if physical availability is 100%, each of the subsequent filters could easily reduce the preceding ratio by a third, resulting in a final or effective coverage of only 20%. Such approaches have been used to assess the coverage of interventions in other areas of health care, e.g. the coverage of mosquito nets^8^ and malaria treatment.^9^

**Figure 1:**
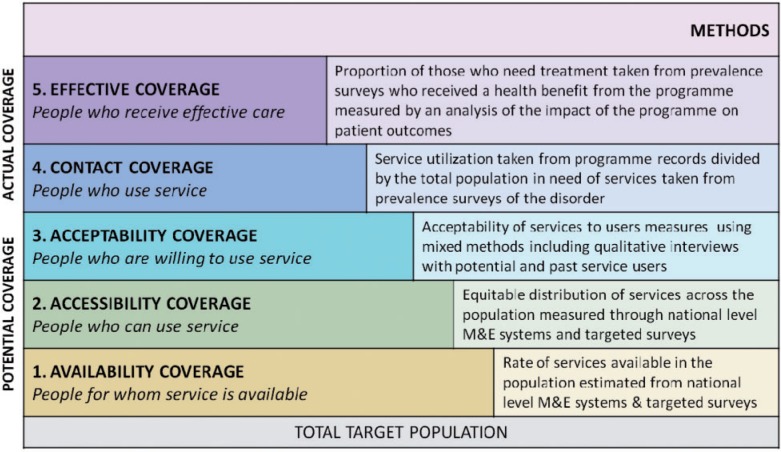
Levels of programme coverage. Figure adapted from Tahanashi 1978.^7^ M&E, monitoring and evaluation

The framework for the definition of coverage used in this review is the five levels of service coverage developed by Tanahashi *et al.*^7^ (Figure 1, modified to include examples of how each level of coverage can be measured). The first three levels address the potential coverage of a programme, comprising the availability of a service, how accessible it is and how acceptable the treatment provided is. For service availability (level 1), a range of tools have been developed and applied in a number of different national contexts for the purpose of comparing the structure, range and supply of specialist adult mental health services in a catchment area. Good examples of these are the WHO ATLAS project^10^ and the WHO AIMS instrument which collects detailed information about the mental health system of a country.^11^

Service accessibility (level 2) is also measured at the health system rather than the individual level and requires a separate set of evaluation methods including national level monitoring and evaluation systems (see e.g. Rotondi 2012^1^,^2^) and targeted surveys (e.g. using the WHO AIMS instrument^11^) to assess how accessible mental health services are to the population in domains such as geographical distribution of services and financial affordability. Acceptability of the services to potential and actual services users (level 3) is most often measured through mixed methods including qualitative interviews with service users. These three types of coverage have been employed only rarely in the field of mental health, and where so, do not in themselves capture the concept of contact or effective coverage as they are pre-conditions of coverage rather than coverage itself.

Levels 4 and 5 of the Tanahashi framework^7^ measure actual rather than potential coverage, and are the focus of this review.

Contact coverage corresponds to level 4 of the Tanahashi framework and captures the proportion of persons in need of a service (e.g. the number of cases with a diagnosable disorder such as schizophrenia or depression) who receive an intervention that is appropriate to their condition. It is calculated as the ratio between the numerator of those receiving services and the denominator of those estimated to need those services in the population. Contact coverage also includes equitable coverage which assesses whether the service covers different socio-demographic or other groups equitably.

Effective coverage corresponds to level 5 and has been defined as ‘the probability that individuals will receive health gain from an intervention if they need it’.^13^ Effective coverage links the three concepts of need, utilization of services and quality of care received, including issues of therapeutic response as well as provider and patient adherence.^14^ Effective coverage therefore places intervention-specific coverage within a health system constraints perspective.

Effective coverage is calculated by assessing the proportion of those in need of a service who gain the intended health benefit from that service. This construct combines contact coverage with intervention effectiveness, the latter measured by tracking changes in clinical outcomes over the course of treatment. The concept of quality of care, for which a number of frameworks have been developed,^15–17^ is central to the concept of effective coverage. For example, the OECD Health Care Quality Indicators Project defines three domains within the concept of quality of care: effectiveness; safety; and responsiveness/patient centredness.^16^ These three domains are essential components in establishing whether an individual receives the intended health gain from an intervention.

In order for the efforts to scale up services for mental disorders to be tracked and evaluated, the extent, equity and effectiveness of the coverage of mental health programmes must be established. Here we describe a systematic review of evaluations of the contact coverage and effective coverage of mental health programmes globally. The results will be used to develop a framework of methods to guide the measurement of coverage linked to efforts to evaluate the scale up of services for mental, neurological and substance use (MNS) disorders.

## Methods

### Search strategy

The methods and results in this paper are presented according to the PRISMA statement for reporting systematic reviews.^18^ A protocol for the review was developed and the search strategy finalized in collaboration with an information scientist. We searched the electronic databases Medline, EMBASE, PsycInfo, Global Health and Econlit in November 2012 using Medical Subject electronic databases using broad search terms designed to capture all evaluations of mental health programmes, as many programmes did not include coverage terms in their title and abstract. The search terms therefore combined the domains mental disorders + service provision + evaluation (for a list of full search terms see Supplementary Appendix A, available at *IJE* online). We manually reviewed the full text of all mental health programme evaluations to select only those which evaluated contact or effectiveness coverage. No restrictions were put on date of publication, but the search was restricted to English language publications due to the size of the literature initially reviewed. Reviews and commentaries were excluded to restrict the search to original reports of programme evaluations.

Grey literature was identified through searching the NHS Evidence and Eldis databases and the websites of relevant non-governmental organizations (NGOs) and multilateral organizations involved in developing and delivering mental health care programmes. In all, 54 individuals in key organizations involved in mental health care delivery and other experts in the field were contacted for additional studies. A Google search using search terms based on the formal search strategy was performed and the search results screened for inclusion until saturation was reached. Finally, a snowball search of potentially relevant references identified through full-text reviews was conducted. Studies identified through this strategy were accepted until March 2013.

### Inclusion criteria

Table 1 lists the inclusion and exclusion criteria for the review. All quantitative evaluations of the contact and/or effective coverage of routine mental health care programmes delivered at scale to the general population were included. Evaluations of population-level access to mental health services were excluded if mental health service utilization was not linked to a specific mental health care programme. Any routine programme designed to treat any mental disorder was included, including single interventions (e.g. psychological therapy), packages of care (e.g. stepped collaborative care for depression) and whole mental health systems (e.g. community mental health teams referring to inpatient units). Time-limited treatment programmes implemented solely as a pilot study or as part of a research project were excluded. Mental health promotion programmes and services delivered to specific populations including veterans, homeless people and prison populations were excluded to focus the review on methods applicable to estimating the treatment coverage of the general population. ‘At scale’ was defined as being delivered in at least one administrative health unit determined according to the country in which the programme was delivered (e.g. in the UK an administrative health unit is defined as a primary care trust).

**Table 1:** Inclusion and exclusion criteria

	Included	Excluded
**Publication type**	Any date	
	Any country	
	English language	Non-English language
	Peer-reviewed articles	Reviews
	Grey literature such as technical reports	Systematic reviews/ meta-analyses
		Commentaries
**Mental health programme**	Any treatment programme for any MNS disorder delivered at scale as part of routine health care. A programme can encompass a single intervention, a package of care or a health system	Mental health care interventions that were only implemented to evaluate their effectiveness as part of a research project or pilot study
		Mental health promotion and mental illness prevention programmes
		Studies evaluating training programmes for mental health care staff delivering treatment interventions
		Web-based mental health treatment programmes
**Study population**	General population including older people, adults, adolescents and children	Specific populations including veterans, prisoners, armed forces and homeless people
**Setting**	All routine health care settings	Specialist settings such as prisons, homes for veterans
**Scale**	Programme delivered at scale defined as at least 1 administrative health unit	Programme delivered to an area smaller than 1 administrative health unit
**Study design**	Any study design reporting the quantitative results of an evaluation of the coverage of a mental health care programme	Programme evaluations which do not evaluate coverage
		Qualitative studies
**Methods**	Study reports the methods used to evaluate coverage	No methods reported or methods reported in insufficient detail
**Outcome**	Measures reported of crude, effective, equitable or population coverage at the individual service user level. These may be reported as ratios, percentages or crude figures	Programmes which do not report a relevant measure of coverage

### Selection of studies

One author of the present study (L.L.) screened the titles of all database search results to remove studies not related to mental health care. Four authors (L.L., D.F., M.D.S. and S.R.) then paired up and independently double-screened the titles of the remaining abstracts and, if necessary, the full texts to determine whether they met the pre-specified inclusion criteria outlined in Table 1. Disagreements were resolved through discussion with a third reviewer.

### Data synthesis

Data were extracted by two authors (M.D.S. and L.L.) using a standard data extraction form including target population, level of evaluation, mental health condition, programme description, study design, the methods used to measure coverage and coverage results. The methods used by included studies to estimate the numerator of service utilization and the denominator of target population were reviewed to provide a description of methods which could be used in programme evaluations.

## Results

**Figure 2:**
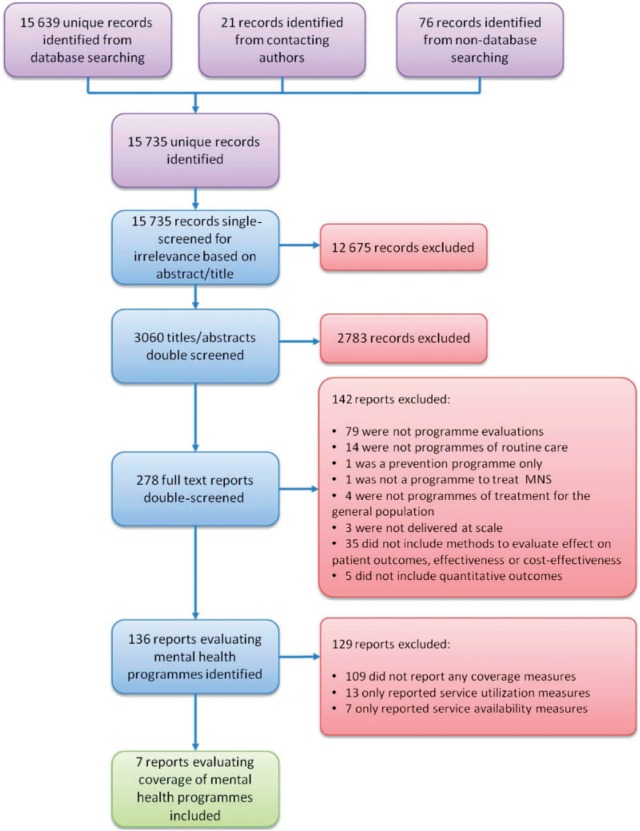
Selection of studies

Figure 2 presents the search and selection process for the review. A total of 15 735 unique records were identified, including 21 from contacting experts in the field and relevant organizations, and 76 from the internet search and references screening. After irrelevant titles were removed, 3060 records were double-screened against the inclusion criteria. Of these, 136 were evaluations of the effectiveness, cost-effectiveness or coverage of mental health programmes. Only 7 of these 136 studies evaluated programme coverage and were included in the review. The other 129 evaluated either the effect of the programme on patient outcomes, or the cost-effectiveness of the programme, and will be the subject of a forthcoming review.

Five of the studies were from high-income countries (four from Europe and one from Australia) and two from upper middle-income countries (Chile and China). Three of the evaluations were of national programmes, two regional and two of single administrative health units (districts). The programmes covered a range of mental disorders including common mental disorders,^19^^,^^20^ severe mental illness,^21^ substance misuse^22^ and a combination of mental disorders.^23–25^ The treatment programmes were equally varied, ranging from: a national programme increasing access to psychological therapies in England;^19^ a regional methadone treatment programme in China;^22^ the nationwide integration of depression treatment into primary care in Chile;^20^ a national financing system to increase access to mental health specialists in Australia;^26^ district-level community mental health services in Italy;^24^^,^^25^ and regional hospital-based services for people with severe mental illness such as schizophrenia or bipolar disorder in Denmark.^21^ Only two of the studies, both government-commissioned reports, evaluated coverage as part of a wider programme evaluation including the effect on patient outcomes and programme costs.^19^^,^^26^ All seven studies included a measure of contact coverage. No study explicitly measured effective coverage, but it was possible to calculate an estimate of this for one study.^19^ Table 2 presents the characteristics of included studies.

**Table 2:** Description of included studies

Study Country Level of evaluation	Condition	Programme description	Study design	Measurement of coverage of coverage	Results
**Coverage of target population of people with MNS disorders**					
Department of Health 2012^1^,^9^ England National	Common mental disorders (CMD)	The Improving Access to Psychological Therapies (IAPT) programme is a large-scale initiative that aims to significantly increase the availability of psychological treatments for depression and anxiety disorders within NHS-commissioned services in England	Government review utilizing IAPT programme dataset which collects performance data on service access, treatment provision and routine patient-reported outcomes	**Crude** *Numerator:* number of patients treated by programme taken from routinely collected programme database *Denominator:* national prevalence of CMD derived from Adult Psychiatric Morbidity Survey. Target set by IAPT to reach 15% of the population with CMD. Based on assumption of number of people with depression and/or anxiety who will seek treatment, number of those who will receive a diagnosis of depression/anxiety, and number of those who will opt for psychological therapy **Equitable** Recovery and access rates analysed by different groups of patients to assess equitable access and outcomes **Effective** Clinical and economic outcomes of patients treated by IAPT are routinely collected by therapists at each appointment session and used as part of the patient’s clinical record as well as collated centrally for programme evaluation	**Crude** Since programme initiation in 2008, average access rates have increased annually, treating over 1.1 million people by 2012. This represents 9.68% of the target the programme set for treating 15% of people in England with CMD annually by 2015. This represents 64.5% coverage of the target population (not calculated by report) **Equitable** Some services do not have representative access from their local communities with regard to age, ethnicity and other factors, but biggest gains in access and recovery are typically among traditionally excluded groups **Effective** 60% of patients completing course of treatment Recovery rates consistently in excess of 45% and approaching those expected from randomized controlled trials. 45 000 people moved off sick pay and benefits The effective coverage rate calculated from these data is 35%
Pirkis 2011^2^,^6^ Australia National	Any MNS	Better Access to Psychiatrists, Psychologists and GPs programme which provides Medicare-based mental health services. The programme started in 2006 and its aim is to improve patient outcomes by encouraging a multidisciplinary approach to mental health care. It does this primarily through the inclusion of a series of new item numbers on the Medicare Benefits Schedule to provide a rebate for mental health services provided	Summative evaluation drawing results from many different studies including analysis of routine data and triangulation of data from other studies (e.g. National Survey of Mental Health and Wellbeing 2007)	**Crude** *Numerator:* number of people receiving services taken from Analysis of Medicare Benefits Schedule data and Pharmaceutical Benefits Scheme administrative data *Denominator:* prevalence of psychiatric disorders from 2007 national Survey of Mental Health and Wellbeing **Equitable** Crude coverage rate broken down by socio-demographic groups	**Crude** Results show a reduction in treatment gap 2006–09, but not 2009–10, indicating a slowing down in reduction of treatment gap. This is broken down by percentage using Better Access services and other health services so proportion of coverage by the Better Access programme can be determined **Equitable** Uptake rates increased most dramatically for those who are most disadvantaged (older people, living in remote areas, living in more socioeconomically deprived areas)
Araya, personal com.^20^ Chile National	Depression	The National Depression Detection and Treatment Programme was launched in 2003 with a network of more than 500 primary care centres. Each centre has a general clinical team composed of primary care doctors, nurses and auxiliary nurses. The programme offers improved case identification, timely and adequate treatment and closely monitored follow up for all enrolled cases	Analysis of existing national cross-sectional community surveys conducted before programme implementation in 2003 and post-implementation in 2009–10	**Crude** *Numerator:* national cross-sectional interview question asking whether respondent had seen a doctor in connection with a depressive episode in the past 12 months *Denominator:* diagnostic tool (CIDI-SF) used to establish 12-month prevalence of depression in national cross-sectional survey **Equitable** Proportion who received treatment broken down by gender and education level	**Crude** The likelihood a depressed individual would have access to health care increased significantly after the programme was introduced [OR 1.87 (95% CI 1.21–2.90)]. **Equitable** Depressed women [41.6% (33.0–50.2) vs 66.3% (58.9–73.7); *P* = <0.001] and those with less education [40.3% (30.0–50.7) vs 66.5% (55.8–77.3); *P* = 0.001] were the primary beneficiaries of the introduction of these universal programmes for depression
Aagard 2004^21^ Denmark Regional	Severe mental illness	Psychiatric system for patients with severe mental ilness in 2 regions. Pre-deinstitutionalization system, which includes hospital inpatient and outpatient treatment	Epidemiological analysis: prevalence study using the national Danish Psychiatric Central Register	**Crude** *Numerator:* rate of inactive patients defined as those not attached to a health service taken from national psychiatric register *Denominator:* prevalence of SMI in the region determined from national register data on the basis of diagnosis of psychosis and high use of psychiatric services	**Crude** Rate of inactive patients is 0.28/1000. Prevalence rate of SMI is 1.31/1000 Crude coverage rate is the inverse of this: 1.31–0.28 = 1.03. 1.03/1.31 = 79% coverage (not calculated in the report)
Lin 2010^22^ China Regional	Drug use disorders	Methadone maintenance therapy programme, established nationwide in 23 provinces, autonomous regions and municipalities and delivered through 558 methadone mainrenance treatment clinics. This paper evaluates the service in a sample of 28 clinics in 2 provinces of China – Zhejiang and Jiangxi	20 service users from each of 28 clinics selected for cross-sectional interviews and urine test. Interviews with 1 service provider from each clinic and analysis of clinic records to collect clinic-level factors	**Crude** *Numerator:* number of service users per clinic taken from clinic medical records *Denominator:* number of opiate addicts registered with the local police department **Predictors of coverage** Characteristics of the 28 clinics included in the evaluation were explored to determine what structural factors predicted coverage levels	**Crude** Crude treatment coverage was 9.1% **Predictors of coverage** Affiliation with local Centres for Disease Control and Prevention, longer opening hours, incentives for compliant clients and comprehensive services were positively associated with higher coverage rates
Martini 1985^24^ Italy AHU (District)	Any MNS	A community mental service including services for those aged under 15 years, walk-in consultation service, rehabilitation service, residential structures,and a psychiatric ward in a general hospital	Continuously collected case register recording all contacts made with services and basic socio-demographic and clinical data	**Crude coverage** *Numerator:* patients registered as being in contact with service from case register *Denominator:* estimation of % of population of catchment area expected to need services based on review of prevalence rates^35^ **Population coverage** Number of patients and rate per 100 000 population aged over 15 years in contact with different parts of the service (inpatient or outpatient) annually for 8 years. Also have disorder specific and total 1-year prevalence rates **Equitable population coverage** Population rate per 100 000 of new episodes of treatment by gender and disorder	**Crude coverage** Ratio between patients and total population of catchment area was between 1% and 1.3%, analogous to the 1–1.5% indicated by review of serious psychopathological disorders treated in outpatient and in hospital settings **Population coverage** Rate of hospital admissions decreased from 139/100 000 in 1974 to 0/100 000 in 1980 (due to closure of hospital). Total 1-year rate of people treated as in- and outpatients was 1313/100 000 **Equitable population coverage** E.g. for schizophrenia 15.55/100 000 for males compared with 20.83/100 000 for females
**Coverage of total population**					
Marinoni 1983^25^ Italy AHU (District)	Any adult MNS	Psychiatric services supplied by the district mental team including an inpatient ward and a community clinic	Continuously collected case register recording all contacts made with services and basic socio-demographic and clinical data	**Population coverage** Rate per 100000 population aged over 15 years in contact with different parts of the service (inpatient or outpatient) annually for 5 years **Equitable population coverage** Population rate per 100 000 of new episodes of treatment by gender and disorder	**Population coverage** Rate of patients admitted to hospital decreases over time and rate of outpatients increases yearly. E.g. 155/100 000 admitted in 1976 compared with 87/100 000 in 1980. 188/100 000 outpatient contacts in 1976 compared with 411/100 000 in 1980 **Equitable population coverage** Treatment rate by gender for different disorders, e.g. new episodes of treatment for schizophrenia 27/100 000 for males compared with 42/100 000 for females

### Methods to estimate contact coverage

#### Estimation of the numerator: service utilization

The studies used three different methods to measure the numerator of service utilization. Five studies determined service utilization from routine patient data collected by the programme.^19^^,^^22^^,^^24^^,^^25^ One of these^21^ collated data collected by the programme to a national level in the Danish Psychiatric Central Register, a computerized register which includes information about all admissions to psychiatric wards and outpatient contacts.^27^ The second method used a national routine database to measure utilization, specifically the Medicare Benefits Schedule which records service utilization through payments made to medical practitioners for services delivered.^26^ The third method, used by the Chile study, used service utilization data not collected by the programme.^20^ This national-level evaluation of the coverage of a recently introduced universal treatment programme for depression in primary care compared independently collected national-level cross-sectional survey data obtained before and after the programme was implemented. Respondents were asked if they had seen a doctor in connection with a depressive episode in the past 12 months. The study therefore estimated the increase in coverage that happened after the national programme was introduced, which may have been due to factors other than the programme itself.

#### Estimation of the denominator: target population

The methods used to measure the denominator, the total population in need of services, varied between studies. Three studies used nationally representative surveys to estimate the prevalence of the disorder,^19^^,^^20^^,^^26^ two of which used regionally disaggregated prevalence figures to provide regional estimates of the target population.^19^^,^^26^ Only one of these studies recognized that not all of those diagnosed with a mental disorder are in need of, or seek, services. A national-level evaluation of the Improving Access to Psychological Therapies (IAPT) programme in England determined the size of their target population based not only on prevalence of the disorder, but on the other factors that determine service utilization.^19^ The authors’ estimated: that of the six million people in England with common mental disorders (CMD) estimated from a national prevalence survey, only half will seek treatment; that a further half will be diagnosed; and that, of these, 70% will opt for psychological therapy. Therefore of the total population of six million people with CMD in England, only 15% (900 000 per year) are likely to actually use psychological therapy and represent the target population that the programme aims to reach in the first 5 years. Programme coverage was estimated as the proportion of this target to which the programme delivered services.

An evaluation of a methadone treatment programme in China used a local register of opiate addicts kept by the police department to estimate the size of the target population in need of treatment.^22^ Although no information was given as to the reliability and validity of these data which have the potential to be highly biased, nor as to its completeness in terms of the proportion of addicts registered with the police, comparing the number of addicts known to local authorities with those receiving treatment is at least a good indicator of the number of people with a known disorder that the programme could realistically aim to reach.

One study used an estimate of the percentage of the population who are expected to need services, based on prevalence rates taken from a review of the global literature^28^ rather than from Italy where the programme was located.^24^ This study, and one other from Italy,^25^ estimate total population coverage whereby the rate of service utilization taken from patient case registers maintained by the programme is calculated per 100 000 of the total population, not the total population of people estimated to have a mental disorder.

#### Methods to estimate equity of contact coverage

Five of the seven studies disaggregated their contact coverage figures by the socio-demographic characteristics of service users such as gender, age and socio-demographic status, in order to estimate equity of contact coverage.^19^^,^^20^^,^^24–26^ However, only the evaluation of the national depression treatment programme in Chile compared the socio-demographic profile of service users with that of the total population of people with mental disorders in order to determine whether access to the programme was equitable.^20^

#### Methods to estimate effective coverage

None of the studies reported effective coverage. The evaluation of IAPT in England was the only study to report the impact of the programme on patient outcomes.^19^ The authors did not use this information to calculate effective coverage, but we were able to calculate this from data presented by the study. In 2012, IAPT treated two-thirds (9.68% out of 15%) of the 900 000 cases of CMD that the programme aims to treat every year. Of these, 60% completed the course of treatment (were adherent), and 45% of these recovered. This is compared with a target recovery of 50% estimated from clinical trials of psychological therapies, thereby requiring a further downward adjustment for the difference between efficacy demonstrated in clinical trials and the effectiveness of the treatment delivered in routine care.^19^ This results in an effective coverage of 35%.

## Discussion

### Main findings

This systematic review of coverage estimates reported by evaluations of mental health programmes has highlighted the paucity of such evaluations, with none at all in lower middle- and low-income countries. As reported elsewhere, there are major gaps in global knowledge of the prevalence of mental disorders, resulting in inadequate information for policy and planning.^29^ Box 1 outlines implications for future research.

**Box 1 Directions for future research**

- Very few evaluations of mental health programmes include estimates of coverage, with no studies found in lower middle- and low-income countries. Current data are inadequate for planning.
- The estimation of contact coverage requires a numerator of routinely collected service utilization data to be compared with a denominator of the number of people in the catchment area who require services.
- Current gaps in national level prevalence data need to be filled by well-conducted population-based surveys in order to provide accurate estimates of the denominator.
- New methods are required to more accurately estimate the proportion of people diagnosed with a mental disorder who are in need of services. This group should form the target population which the programme aims to cover, not all those diagnosed with a mental disorder.
- Estimates of coverage should be broken down by factors such as gender and socioeconomic status to determine whether programme coverage is equitable.
- The estimation of effectiveness coverage involves adjusting the contact coverage of a programme by the effectiveness of the programme on patient outcomes, to determine the proportion of those in need of services who benefit from them.
- Evaluations of mental health programmes in all settings should routinely incorporate measures of contact and effectiveness coverage to improve existing services and to inform efforts to scale up services in settings where there are none.

This lack of evidence is partly due to the methodological difficulties of estimating contact coverage. To estimate the denominator of the population in need of services in a catchment area, estimates of the prevalence of the disorder in the catchment population are needed. Collecting this information is beyond the scope of individual programmes, so routine data need to be utilized. In high-income countries, prevalence data are sometimes available through routine national surveys such as the Adult Psychiatric Morbidity Survey in England^30^ and the national Survey of Mental Health and Wellbeing in Australia.^31^ In settings where national- or regional-level survey data may not be available, reference can be made to the estimated prevalence of mental disorders in the scientific literature, for example in the World Mental Health surveys^32^ and the national prevalence rates for different disorders calculated as part of the Global Burden of Disease 2010 study.^33^ However, these prevalence data are patchy, with more complete prevalence data for North America and Australasia, highly variable data from Europe, Latin America and Asia Pacific and poor levels of data in other regions of Africa and Asia.^29^ In addition, the prevalence estimates that do exist are often for specific regions of a country, sub-groups of the population or specific disorders, and may not represent the population which a specific programme seeks to serve. If no such prevalence estimates for the population exist, then the number treated can be compared with the total population size to provide a ratio of the number treated per 100 000, which remains an informative metric for planning services, tracking changes in service utilization over time and comparing programme coverage with other non-mental health programmes, though these cannot provide an estimate of the proportion of those in need who are receiving services.

Furthermore, the methods for measuring the denominator for coverage are based on the assumption that all those who are diagnosed with a mental disorder are in need of treatment. This assumption clearly needs to be tested as severity of illness, suitability of treatment provided by the programme, other sources of support available and patient preferences will all affect the need for or uptake of services. Only one study included in this review addressed this issue. The UK IAPT study^19^ determined the size of their target population based not only on the prevalence of the disorder, but on the other factors that determine service utilization. Whereas using such a ‘rule of thirds’ to estimate those that the programme should realistically intend to cover is crude, it does at least reflect the fact that a programme should not attempt to cover all those with the target disorder in the population it serves, but rather the fraction comprising those who are willing to receive and would benefit from the treatment provided by the programme.

Measurement of the number of people using the service (numerator) requires routine monitoring data to be collected by programmes. We anticipate that most programmes could routinely collect these in the form of numbers of patients referred and treated. To assess the equity of coverage, basic socio-demographic information is also needed such as age, gender, area of residence and ethnic group, ideally broken down by diagnosis. Such information can be routinely collected by Health Management Information Systems (HMIS), but it needs to be compared with data from community-based prevalence surveys to determine whether programme coverage is equitable compared with the socio-demographic profile of the target population. In addition, population based surveys which include questions on treatment received can provide estimates of service utilisation, if the treatment is ascribed to particular programmes.

The estimation of effective coverage requires outcome data for those treated, which are much more intensive to measure. It is notable that none of the included studies reported effective coverage, though we were able to calculate this for one of the studies based on the data provided by the IAPT programme.^19^ However, many programmes in high-resource settings do routinely collect patient outcome data as part of their clinical records, and similar systems can be implemented in low-resource settings with minimal investment and staff training. Examples include routine clinical information systems being implemented in community mental health programmes in Nigeria by the NGO CBM, and by the NGO Health Net TPO in Burundi, Indonesia, Nepal, Sri Lanka and Sudan.^34^

To be of further use for service planning and development, evaluations of programme coverage should include an analysis of the determinants of coverage, so that factors which increase coverage can be built into the design of programmes. Only one of the evaluations included in this review^22^ estimated differences in coverage between treatment clinics and explored the reasons for these differences. The study showed that structural factors such as the length of opening hours and incentives for compliant clients were positively associated with increased coverage. Such information, especially if incorporated into pilot evaluations of new programmes, could be used for service development and improvement.

There is a separate but related literature on the ‘treatment gap’ which seeks to estimate the lack of coverage of any form of treatment for specified mental health problems.^2^^,^^22^ The denominator is estimated in the same way, that is by using nationally representative community surveys to estimate the total number of people with mental disorders in the population (as determined by a diagnosis of a mental disorder using a screening or diagnostic tool). Concerning the numerator, for treatment gap studies this is the number of cases in need who receive no care or treatment for their condition, which is typically measured via cross-sectional community surveys that ask respondents to recall any treatment for a particular disorder for which they screen positive in the survey. Such survey data do not yield information about the coverage or effectiveness of specific interventions or programmes, thus the treatment gap differs from the direct measurement of coverage associated with a specific programme. In short, repeated measurement of the treatment gap can inform policy and planning by revealing changes in the proportion of people receiving no care, but needs to be supplemented with information on the effective coverage of individual programmes so as to determine which types of programmes produce the best and most equitable patient outcomes for the largest numbers of people.

### Strengths and limitations of the review

There are a number of limitations of the review. First, as the term ‘coverage’ was so rarely used in descriptions of programme evaluations, we did not use specific search terms for coverage but rather manually searched all programme evaluations to determine whether they included coverage. Due to the scope of this exercise (56 000 studies located in a search not restricted by language) we had to restrict the review to English language publications. Second, we did not list individual disorders by name. This may have resulted in some evaluations of treatment programmes which are not consistently reported as mental disorders being missed (such as substance misuse or eating disorders). To mitigate these limitations, we used a wide-ranging search strategy including electronic database and internet searches and contacting key organizations and experts in the field to identify both published and grey literature. We used broad search terms to capture the wider literature on mental health programme evaluation, and then hand-searched these results to locate evaluations of coverage. We are confident that the studies included in this review represent the scope of the English language literature on this topic.

## Conclusion

Evaluating the contact coverage and the effective coverage of mental health programmes is essential in order to track efforts to scale up effective and equitable services for people with mental disorders, and has great potential to guide work to improve the quality of existing services. Estimates of contact coverage could be incorporated into the routine monitoring of mental health programmes simply and cheaply by analysing data routinely collected by programmes and comparing these with population prevalence estimates from national surveys. Better estimates of programme contact and effective coverage, the equity of coverage and the factors that affect coverage are needed to ensure that there is optimal investment of scarce resources into existing mental health programmes, and that new mental health services implemented in low-resource settings are designed to optimise the effective and equitable programme coverage of those in greatest need.

## Supplementary Data

Supplementary data are available at *IJE* online.

## Funding

This work was supported as part of the PRogramme for Improving Mental health carE (PRIME), funded by the UK Department for International Development (DfID) for the benefit of low- and middle-income countries (HRPC10). M.D.S. is funded by an LSHTM/Wellcome Trust Fellowship, L.L. by Grand Challenges Canada (EPPHVK29), D.F. by the National Institute of Mental Health (0178-04), S.R. by the UK Department for International Development (HRPC10) and D.C. is partly funded by the UK Department for International Development (HRPC10), Joanna Schellenberg by the Bill and Melinda Gates Foundation and Vikram Patel by a Wellcome Trust Senior Research Fellowship in Clinical Science.

The funders had no role in study design, data collection and analysis, decision to publish, or preparation of the manuscript.

## Supplementary Material

Supplementary Data
