# Supplementary material for: Estimating the coverage of mental health programmes: a systematic review
Source: Int J Epidemiol. 2014 Apr 22;43(2):341–53. doi: 10.1093/ije/dyt191 (PMC3997372; doi:10.1093/ije/dyt191)
Supplement: Supplementary Data [file supp_43_2_341__index.html]

Supplementary Data 

# Estimating the coverage of mental health programmes: a systematic review

## Supplementary Data

files

**Files in this Data Supplement:**

- Supplementary Data - pdf file
